# Irrigation and debridement for knee osteoarthritis patients with suspected infection by intra-articular injection before total knee arthroplasty: a retrospective study

**DOI:** 10.1186/s13018-022-03054-z

**Published:** 2022-03-24

**Authors:** Haochen Jiang, Hengfeng Yuan, Hai Hu

**Affiliations:** 1 Department of Orthopedic Surgery, Shanghai Jiao Tong University Affiliated Sixth People’s Hospital, 600 Yishan Road, Shanghai, People’s Republic of China; 2 Department of Orthopedic Surgery, Xuhui Branch of Shanghai Jiao Tong University Affiliated Sixth People’s Hospital, Shanghai, People’s Republic of China

## Abstract

**Background:**

Patients suffer from knee osteoarthritis (KOA) pain may seek for intra-articular injections before total knee arthroplasty (TKA), which have a possibility of causing the joint sepsis. However, the management and clinical outcomes of these patients following TKA remain uncertain.

**Methods:**

Patients with a history of intra-articular injection, in which a joint sepsis was suspected, were included. The patients received joint irrigation and debridement (I&D) and antibiotic treatment until serum inflammatory indicators returned to normal level before TKA. The information of joint fluid routine and culture, synovium section and culture, and serum inflammatory indicator values were collected. Range of motion, Knee Society Scores (KSS) and Western Ontario McMaster Universities Osteoarthritis Index (WOMAC) were used for functional evaluations.

**Results:**

A total of 17 patients with 17 knee joints were included, all with elevated C-reactive protein (CRP) levels (23.5 ± 8.7 mg/L) as well as increased number of white blood cells (WBC) in the aspiration (50.8 ± 15.3) × 10^9^/L, but no positive cultures were found. The culture of synovium detected three positive results: two *Staphylococcus epidermidis* and one *S. aureus*. I&D treatment had no obvious effect on the functional outcomes of KOA, but alleviated the joint pain (*p* < 0.01). Furthermore, we found that I&D pretreatment could increase the operation time with about 10 min longer than the primary TKA (*p* < 0.01). With respect to TKA outcomes, I&D had a slight influence on the knee flexion (*p* < 0.01), but no significant difference was identified between the two groups for KSS and WOMAC (all *p* values > 0.05). In addition, there was no significant difference in complication rates between the two groups in the last follow-up.

**Conclusion:**

I&D treatment is a valuable procedure for suspected knee infection, which has a higher incidence of detecting microorganisms while does not influence the functional outcomes and complication rates of TKA. However, further larger studies are required to confirm these findings.

## Introduction

Intra-articular injection is a common practice used by many orthopaedic surgeons for the conservative treatment of knee osteoarthritis (KOA), which can provide effective short-term pain relief for the patients [1, 2]. Reports have shown that approximately thirty per cent or more of KOA patients had received at least one injection before they were diagnosed for the requirement of total knee arthroplasty (TKA) [3]. Although organizations including the American College of Rheumatology [4], American Academy of Orthopaedic Surgeons [5] and Chinese College of Rheumatology [6] recommend the corticosteroid and viscosupplementation injections, a large database study reported that patients receiving injections before TKA are at higher risk for post-operative infection [7].

The management for these patients demanding TKA is challenging if they are suspected with native joint infections, especially confirming the presence of pathogens in the joints is difficult. The joint fluid test is not always dependable to exclude pre-operative presence of pathogens due to high false-negative rates, as well as the amount of microorganisms could be too few to be detected with routine joint fluid culture [8]. The synovial fluid leukocyte count and differential is helpful for diagnosis of knee infection but not in detecting the presence of microorganisms [9]. Recent report has shown that intra-operative synovial tissue culture could be the most valuable inspection to diagnose this condition other than pre-operative blood test and joint fluid examination [10]. Although treatment for prosthetic knee infection has been well established [11], few studies address the treatment programs and outcomes for patients with suspected native knee infection prior to TKA.

In the present study, we performed irrigation and debridement (I&D) treatment for KOA patients with suspected knee infections after joint injections. Then, we compared the intraoperative data, following the functional outcomes and complications of these patients after TKA with primary TKA patients. We aimed to testify whether I&D is a cost-effective treatment for these patients.

## Patients and methods

### Patient recruitment

We retrospectively identified KOA patients who underwent I&D between 1 March 2014 to 31 January 2017 with suspected infection after intra-articular injection. The knee infection was suspected based on increased inflammatory parameters in serum or elevated WBC count in synovial fluid after enrolment. Eligible patients were participants with radiologically established KOA who met American College of Rheumatology Criteria. The following exclusion criteria for this study were considered: (1) rheumatoid arthritis or other autoimmune diseases that may increase infection rate; (2) previous surgery or infection history of the affected knee before intra-articular injection; (3) use of antibiotics for any reason 3 weeks prior to I&D; and (4) refusal to be included in this study. This study was approved by our institution’s ethical committee, and informed consent was obtained from each patient before participation.

For each patient included in this study, the injection history was also recorded including: injection type (corticosteroid and/or viscosupplementation), injection times, and the time between first injection and debridement surgery. The joint fluid routine and culture, inflammatory parameters in serum (C-reactive protein, CRP) and WBC count in synovial fluid were examined as well. In addition, each patient was paired with KOA patient by age, gender, body mass index (BMI), alcohol, tobacco and comorbidities. The KOA patients for comparison had normal or negative results for each laboratory test as mentioned above. The surgical time and intraoperative blood loss were recorded.

### Management

While the joint infection was suspected, I&D was performed by the same experienced surgeon. The operated knee was cleaned with soap and sterilized with iodophor before the antimicrobial incise drape was draped. The standard surgical approach was used as middle skin incision and medial parapatellar arthrotomy. All synovium was excised, and four pieces of synovium were obtained separately from the suprapatellar capsule, intercondylar notch, medial groove and lateral groove for frozen pathological section, routine pathological section and culture. Pulsed lavage was used before incision suture. Prophylactic antibiotics (cefotiam 1.0 g and levofloxacin 0.2 g) were administered two times to each patient before the culture result of the synovium was known. If the result was negative and serum parameters returned to baseline, the following TKA was planned. However, if the patient had positive culture result, at least 6 weeks of sensitive antibiotics were given, and then TKA would be taken into consideration until serum parameters were normal.

TKA was performed by the same surgeon with the same surgical procedure above in both I&D group and paired group.

### Outcomes

The patients were followed up for a minimum of 12 months, and post-operative complications such as infection and deep vein thrombosis were noticed at any point during follow-up. The infection was defined according to the guidelines of the Centers for Disease Control and Prevention [12]. Functional outcome measures of the patients were collected pre-operatively and post-operatively consisting of range of motion (ROM), Knee Society Scores (KSS) and Western Ontario McMaster Universities Osteoarthritis Index (WOMAC). The outcomes were collected by use of a designed form at the 6-week, 12-week and last follow-up visits.

### Statistical analysis

The statistical analyses were performed using SPSS version 17.0 software (SPSS Inc., Chicago, IL). The continuous data were presented as mean ± standard deviation. The chi-square test was used for qualitative variables comparison, and *p* value < 0.05 was considered as statistically different.

## Results

A total of seventeen patients with seventeen knees were included for suspected knee infection after intra-articular injection. The synovial fluid WBC count was (50.8 ± 15.3) × 10^9^/L, and ten patients were higher than 50 × 10^9^/L. Thirteen of the patients had knee joint pain, and fifteen had swelling joints for more than 3 weeks. All patients had elevated inflammatory parameters in serum, with CRP value 23.5 ± 8.7 mg/L. The number of injections among the participates was 5.4 ± 2.4. In all joint synovial fluid specimens, cultures were negative. However, three patients with three knees had positive intra-operative synovial tissue culture results: two *Staphylococcus epidermidis* and one *S. aureus*.

With regard to the functional outcome measures, the scores were not significantly different before and after I&D surgery, except for the WOMAC pain scores, with the value decreased from 24.1 ± 2.5 to 20.5 ± 2.6 (*p* < 0.01; 95% confidence interval 1.73–5.32).

**Fig. 1:**
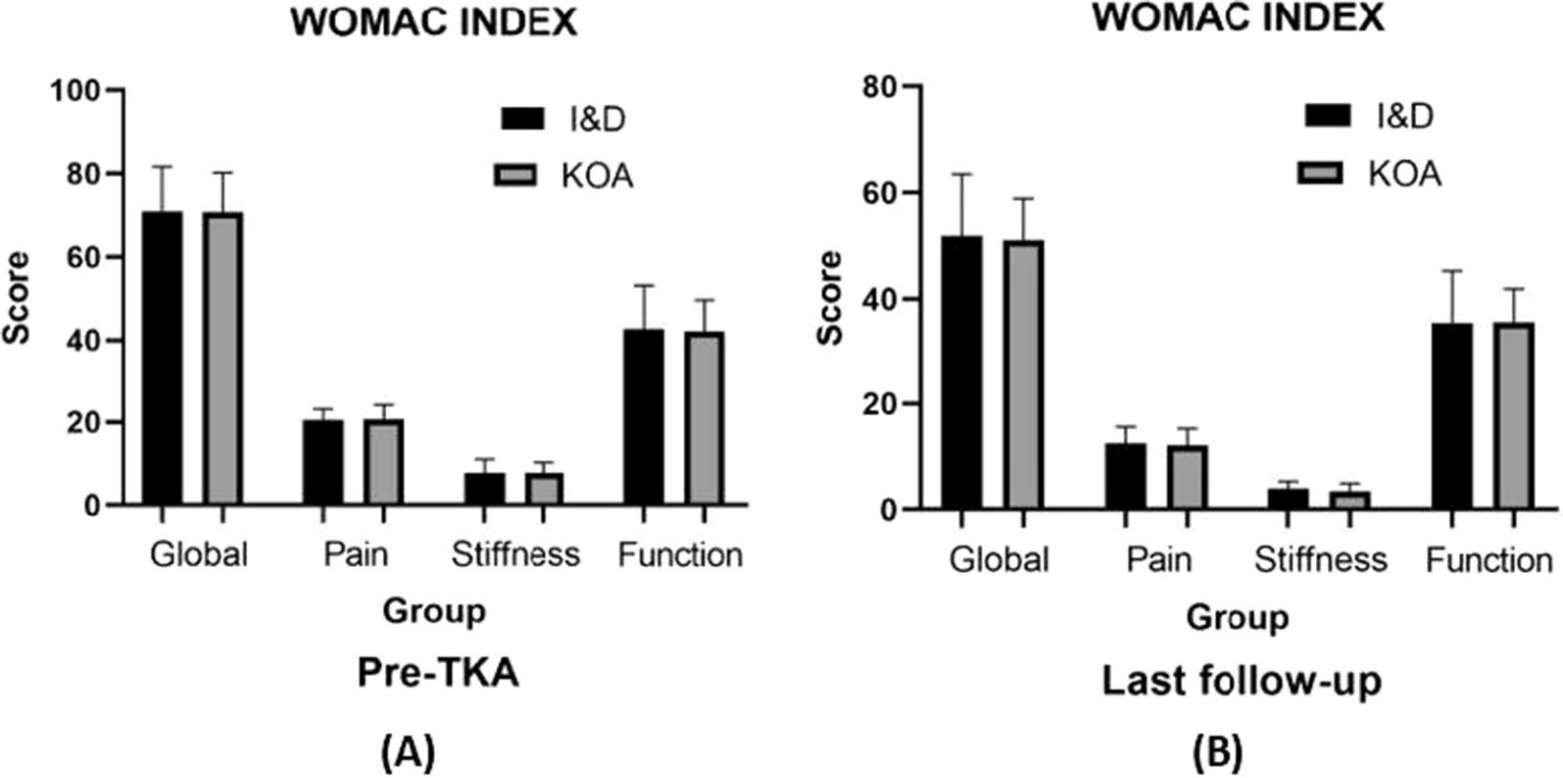
Comparison of WOMAC score between I&D group and KOA group. The WOMAC score of pre-TKA between I&D and KOA (**A**). The WOMAC score of last follow-up between I&D and KOA (**B**)

There were no significant differences with respect to the functional measures between patients after I&D treatment and the matched KOA patients in Table 1. The mean operating time showed a statistically significant difference between TKA after I&D group and primary TKA group, with 86.0 ± 2.9 min versus 77.0 ± 4.7 min (*p* < 0.01; 95% confidence interval -12.00 to -5.86). However, the volumes of blood loss were not significantly different, with 25.2 ± 4.3 ml versus 22.9 ± 3.4 ml (*p* = 0.094). After a mean follow-up of 24.2 months, the post-operative ROM of flexion was significantly poorer in the TKA after debridement group than in the primary group, with 102.2 ± 8.3 versus 112.0 ± 9.1 (*p* < 0.01; 95% confidence interval 4.41–15.19) in Table 2. However, ROM of extension showed no significant difference. With regard to the scores of KSS and WOMAC, there was no significant difference between the groups (all *p* values > 0.05) in Fig. 1 and Table 2. Finally, none complications were reported in both of the groups.

**Table 1:** Baseline characteristics of the I&D group and KOA group

Parameters	I&D group	KOA group
No. of patients	17	30
Age (y)	64.2 ± 9.2	65.0 ± 7.2
Gender (female/male)	10/7	17/13
BMI (kg/m^2^)	27.5 ± 3.3	27.7 ± 3.3
Alcohol	8	14
Tobacco	9	16
Comorbidities		
Diabetes	5	12
Hypertension	10	25
Urinary infection	2	3

*I&D* irrigation and debridement, *KOA* knee osteoarthritis

**Table 2:** Comparison of clinical outcomes between I&D group and KOA group

Variables	I&D group	KOA group
*Pre-TKA*		
KSS score	110.5 ± 27.9	119.7 ± 26.9
WOMAC score		
Global	70.9 ± 10.7	70.7 ± 9.5
Pain	20.5 ± 2.6	20.7 ± 3.4
Stiffness	7.7 ± 3.3	7.7 ± 2.6
Function	42.7 ± 10.4	42.2 ± 7.4
Range of flexion	93.5 ± 8.1	94.8 ± 7.7
*Last follow-up*		
KSS score	172.3 ± 11.0	172.2 ± 12.3
WOMAC score		
Global	51.9 ± 11.6	51.1 ± 7.8
Pain	12.5 ± 3.1	12.1 ± 3.2
Stiffness	3.9 ± 1.4	3.4 ± 1.5
Function	35.5 ± 9.8	35.6 ± 6.3
Range of flexion*	102.2 ± 8.3	112.0 ± 9.1

*TKA* total knee arthroplasty, *I&D* irrigation and debridement, *KOA* knee osteoarthritis, *KSS* Knee Society Scores, *WOMAC* Western Ontario McMaster Universities Osteoarthritis Index

*Statistically significant (*p* < 0.01)

## Discussion

Periprosthetic joint infection (PJI) is a disaster complication after TKA which is associated with substantial patient morbidity [13, 14]. Although the correlation between intra-articular injections and the risk of post-operative infection following TKA is still controversial, the most serious complication from injections is joint sepsis [15, 16]. To our knowledge, few reports have mentioned the management of joint infections caused by injections prior to TKA.

I&D treatment has been used for TKA PJI for a long time [17]; however, recently a multicentre study showed that I&D had a high failure rate with 5-year mortality of 19.9%, and this treatment had limited ability to control joint infection subsequent to TKA and should be used selectively under optimal conditions [18]. However, our study indicated that I&D is useful for infections prior to TKA. Although I&D could not improve the ROM or functions of knee joint, it could relieve the joint pain, which may be due to removal of inflammatory synoviums.

Our results have shown I&D could increase the operating time with about 10 min longer than in the primary TKA. We observed that during the performance of TKA, I&D could cause a second-stage tissue adhesion and make the exposure more different. With respect to ROM, we found that I&D had a slight influence on the flexion of the knee joint. However, I&D did not lead to the short-term functional outcome differences when compared with primary TKA. Moreover, in our study, we find that I&D prior to TKA was not associated with increased rates of complications and infection. Therefore, we strongly recommend the use of I&D treatment for the infected knees prior to TKA.

This study has several limitations. Firstly, the number of participants included was too small to accomplish a more statistically confirmative conclusion. Secondly, the patients with primary TKA were not randomized because we wanted to closely match them for various other demographic factors of patients after debridement. However, one of the strengths of this study was that all operations were performed by the same surgeon exclusively using the same approach. Finally, the follow-up time for these patients is too short, while the post-operative outcomes and complications need more time to evaluate the efficacy and safety of debridement prior to TKA.

In conclusion, intra-articular injections for KOA had a possibility of causing joint infection, and culture of synovium tissue obtained from debridement had a more positive rate compared with other methods. Although debridement could not significantly improve the joint functions and increase the operation time when performing TKA, it could guarantee the safety of TKA and avoid the chance of revision surgery due to PJI. Moreover, the clinical outcomes and complications of TKA after debridement showed no significant differences compared to primary TKA.

## Data Availability

The data sets generated and/or analysed during the current study are not publicly available due to the data application regulations of the hospital but are available from the corresponding author on reasonable request.

## Abbreviations

KOA: Knee osteoarthritis
TKA: Total knee arthroplasty
I&D: Irrigation and debridement
ROM: Range of motion
KSS: Knee society scores
WOMAC: Western Ontario McMaster University Osteoarthritis Index
CRP: C-reactive protein
*S. aureus*: *Staphylococcus aureus*
WBC: White blood cell count
PJI: Periprosthetic joint infection
BMI: Body mass index
